# On the Profession of Dental Surgery

**Published:** 1876-12

**Authors:** John Tomes

**Affiliations:** London.


### ARTICLE V.

### *On the Profession of Dental Surgery.*

A Letter addressed to the Editor of the Lancet.

BY JOHN TOMES, ESQ., F. R. S., LONDON.

Sir: The attempt to bring the influence of the medical profession to bear upon the training of dental surgeons can be of advantage only when medical men are made fully acquainted with the scope and requirements of dental surgery, an accurate knowledge of which they do not at present possess. The existence of special journals, while they render great service in bringing before the dental practitioner all that relates to his calling, tends to remove from the eye of the general practitioner much that he well might know, and more that it is needful that he should know, if he would form a just opinion of the present state of dental surgery. The absolute necessity for a long special training in order to acquire moderate capabilities as a dental practitioner is very generally overlooked, and the power to pass an examination in surgery and to write a prescription is accepted as a sufficient education for the dental surgeon. But those whose powers constitute them efficient judges know full well that there is little difficulty in recognizing what is required in the treatment of a case, but very great difficulty in acquiring the power to carry it into effect. To take out a large portion of the softened tissue from a carious tooth and to fill up the hole with a plastic material requires little skill; but to remove the diseased part as fully as may be, to leave the cavity in a form capable of retaining a long-lasting plug, and to introduce that plug—in fact, to perform the operation up to the highest standard of excellence, without an undue expenditure of time exhaustive to the patient and operator—requires great skill, a degree of skill gained only by patient training over at least two years in pupils naturally not inapt. Let any one interested visit the dental hospital, and watch the performances of students of one year then those of two years' training, and, if possible, the operations of the more skillful teachers; let him then try his own hand, and all I have stated will be admitted. That which is true respecting filling teeth applies also to other dental operations. But the dental surgeon will be occupied three-fourths of his time in treating carious teeth, and if his operations are rightly performed, he will have rendered good service, and secured the lasting gratitude of his patients.

Were it otherwise we should find medical practitioners using the leisure hours of their early professional life in the performances of dental operations. In truth, the education of the surgeon does not embrace dental surgery. A man may be a member of the College of Surgeons or Physicians or of the Apothecaries' Company, and yet know nothing of dental surgery; and it would be unreasonable, and consequently, all but impossible, to impose upon him a long and expensive training in a specialty he is not specially destined to practice; and, again, the knowledge acquired would be speedily lost unless constantly practiced. That which would be true of the general surgeon applies with equal force to the dental surgeon, whose knowledge of general surgery fades from disuse by the time he has acquired his general diploma; for his specialty will, if rightly practiced, demand all his time and thoughts. He cannot remain an effective general surgeon if he would be a faithful dental surgeon.

He may be a legally qualified practitioner, but, without use the knowledge he acquired up to the level of a pass examination will speedily waste, and he will become morally disqualified.

These facts were fully recognized twenty years ago, when the College Surgeons instituted its department of dental surgery, laid down a curriculum to be followed by students, and after examination granted a diploma of fitness to practice, under the title of Licentiate in dental surgery. In this curriculum certain subjects embraced in the general medical education, but scarcely needful to the Dentist, such as midwifery, forensic medicine and botany, etc., were left out, and in their stead special dental subjects was introduced, leaving the time and cost of the profession strictly unaltered. The wisdom of this course is shown in the fact that the dental school formed to meet the requirements of the curriculum has from year to year increased the number of its students, who at the present time nearly approach a hundred; and that the education meets the necessities of the dental surgeon is proved by its voluntary acceptance by those whose purpose it is to follow dental practice. For it must not be forgotten that the legal powers given to the colleges in the dental charter are permissive only; hence the education is offered, not enforced. The value of the diploma has been steadily advanced by extending the educational opportunities, by gradually increasing the strictness of the examination, and by the addition of writing to the *viva voce* questions. And the preliminary examination in arts now required of the medical students before entering upon professional studies will also be required of the dental student from October, 1877.

The licentiateship is the qualification needed by the dental surgeon, for no other indicates that the possessor is practically acquainted with dental surgery; and surely, if we would gain our living by dental practice, we must before all else be skillful dentists. I use the word *dentist* because the public will call us dentists, do what we will, just as they call the ophthalmic surgeon an oculist; and if we suffer, it will not be by the name but by our want of worth.

I do not for one moment argue against the dental student taking a general medical qualification; on the contrary, I would urge the student whose means and time will allow, to take the membership or fellowship of the College of Surgeons or Physicians, and I would further advise him to take the B. A. of one or other of our Universities. But I do protest against the student making a general qualification stand in the stead, and take the place, of the special one, if he proposes to gain his living by our specialty.

Dental surgery some years back suffered greatly from one or other of two conditions. The practitioner had at the outset only a general medical education, or in many cases he possessed no education at all, and the suffering is not yet at an end; but the wise course taken by the College of Surgeons has already very greatly reduced the evil, and will doubtless ere long, to the exclusion of the incompetent, provide the public with a very efficient race of practitioners.

It is reasonably hoped that the permissive education, the success of which no well informed person can deny, will become compulsory, and that a distinctive line will be drawn between the competent and incompetent practitioner, recognizable by the public.

The membership and licentiateship jointly may be said to meet the case, but they involve a higher and more costly education than either taken singly, and it is not reasonable to expect that all dental students will be prepared to meet the demand of the double qualification. For such, and they will probably form the majority, at least for some time, the licentiateship will suffice, as being the educational equivalent of the single qualification in the general practitioner.

There has been much loose talk about the status of the dental practitioner. We had better work more and talk less upon the education of ourselves and our fellows. The Legislature can neither give nor withold social status. The public will settle the question, and our position will be determined by our individual and collective professional and general culture, by our professional usefulness and scholarly attainments.

I am, Sir, yours truly,

June 14th, 1876. JOHN TOMES.

—*Johnston's Dental Miscellany.*

---

